# Breastfeeding factors and gut microbiota dynamics in infants during the first year: a Beijing cohort study

**DOI:** 10.3389/fnut.2026.1930295

**Published:** 2026-09-07

**Authors:** Xinran Liu, Qianying Guo, Mingxuan Cui, Xuening Li, Chen Yang, Shilong Zhao, Lina Pan, Xiaoyu Peng, Linlin Wang, Peng Liu

**Affiliations:** 1 Department of Clinical Nutrition, Peking University People's Hospital, Beijing, China; 2 Department of Obstetrics and Gynecology, Peking University Third Hospital, Beijing, China; 3 Institute of Reproductive and Child Health/National Health Commission Key Laboratory of Reproductive Health, School of Public Health, Peking University, Beijing, China; 4 Ausnutria Dairy (China) Co., Ltd, Changsha, China

**Keywords:** breast milk composition, breastfeeding, dietary balance index, infant gut microbiota, maternal diet

## Abstract

**Objective:**

To clarify the impact of maternal nutrition and breastfeeding on infant gut microbiota development.

**Methods:**

In a cohort of 67 mother-infant dyads, maternal diet and milk composition were analyzed alongside longitudinal infant fecal 16S rRNA profiling at 42 days, 6 months, and 12 months.

**Results:**

The infant gut microbiota underwent marked shifts over the first year, with increases in *Firmicutes* and *Bacteroidota*, decreases in *Actinobacteriota* and *Proteobacteria*, and increasing alpha diversity. Feeding mode was the strongest predictor of infant gut microbiota. Feeding mode, delivery mode, maternal dietary deficiency, complementary feeding timing, and breast-milk density were each associated with distinct microbial taxa, with 14 genus-/phylum-level associations identified.

**Conclusion:**

Significant phylum-level shifts occurred over the first year, specifically increases in *Firmicutes* and *Bacteroidota*. Feeding mode was the dominant predictor of microbiota composition at 12 months, with additional associations for delivery mode, complementary feeding timing, maternal dietary deficiency, and breast-milk density.

## 1 Introduction

Infant gut microbiota establishment in early life is a determinant factor for lifelong health, which is dynamically modul ated by multiple perinatal and postnatal environmental factors (1).

As the dominant modulator of early infant gut microbiota maturation, breastfeeding supplies unique nutrients and bioactive components that drive the colonization of beneficial gut bacteria, and exclusive and sustained breastfeeding is well-recognized to shape a healthy intestinal microecology in infants (1, 2).

First, maternal diet modulates breast milk nutritional properties and microbial composition, thereby indirectly programming the infant gut microbiome; probiotic-rich maternal diets have been verified to enrich beneficial bacteria in both breast milk and infant intestines. Notably, the lack of standardized maternal dietary evaluation methods limits relevant population studies. To address this issue, the present study adopts the Dietary Guidelines for Chinese Residents and the Diet Balance Index (DBI) for quantitative and standardized assessment of lactating maternal dietary patterns (2).

Second, the composition of breast milk itself plays a crucial role in developing the infant gut microbiome. Components such as human milk lactose and oligosaccharides (HMOs) act as prebiotics, promoting the growth of beneficial bacteria like Bifidobacteria (3). Other elements, including antimicrobial proteins and immune cells, help protect against harmful bacteria and support a healthy gut microbiota.

Third, breastfeeding duration determines the duration of infant exposure to breast milk beneficial components, with prolonged breastfeeding contributing to higher diversity and greater stability of the infant gut microbiota (4).

Evidently, it is essential to consider the influence of geographic and ethnic factors on the infant gut microbiome. Regional and racial differences can significantly impact microbiome composition, making it crucial to conduct research within specific populations (5). Despite extensive studies in various parts of the world, there is a notable lack of research on this topic in China. This gap highlights the significance of this study, as it aims to provide valuable insights into the relationship between breastfeeding and the infant gut microbiome in the Chinese context.

In conclusion, understanding the intricate relationship between breastfeeding and the infant gut microbiome necessitates a comprehensive examination of maternal diet, breast milk composition, and breastfeeding duration. These factors collectively influence the establishment and development of the infant gut microbiota, thereby playing a critical role in shaping the child's future health outcomes. This research aims to unravel the complex interactions within this triad in order to optimize breastfeeding practices and improve infant health.

## 2 Materials and methods

### 2.1 Subjects

This study utilized a follow-up mother-infant cohort from Peking University People's Hospital. Screening and enrollment were based on the following inclusion criteria: full-term infants born at the hospital, mothers aged 20–45 years, and either exclusively or predominantly breastfeeding (breastfeeding constituting over 80%). Infants were excluded if they had been administered antibiotics or probiotics.

In total, 33 male infants and 34 female infants were included in the study; all infants were singletons. Ethical approval was obtained from the Ethics Committee of Peking University People's Hospital (Approval Number: 2020PHB113-01), and informed consent was secured from all participating mothers.

### 2.2 Dietary patterns of lactating mothers

The dietary status of lactating mothers was evaluated using the DBI method (6). To enhance accuracy, participants were provided with food illustration diagrams which displayed the visual representation of different foods in various weights and portion sizes (7). These visual aids helped participants to better estimate the quantities of food they consumed by providing a clear and intuitive reference. This evaluation was conducted based on complete 3-day 24-h dietary recall data. The DBI indicators include the Total Score (TS), which reflects the average overall dietary quality; the High Bound Score (HBS), indicating the degree of excessive intake in the diet; the Low Bound Score (LBS), reflecting the extent of dietary deficiency; and the Diet Quality Distance (DQD), representing the imbalance in the intake of various food categories. First, the average daily intake of each food group for individuals was calculated. Subsequently, using the DBI scoring method, each participant's DBI scores were determined, including TS, HBS, LBS, and DQD.

### 2.3 Composition detection of breast milk

On the morning of the 42nd day postpartum, 5 ml of breast milk was collected from a single breast using either an electric breast pump or manual expression. The collection occurred mid-feed, approximately 5–7 min after the start of lactation. The exact collection time was recorded, and the samples were immediately frozen and transferred to a −80 °C freezer for further analysis.

The breast milk was analyzed for lactose, fat, protein, energy, minerals, and water content using a human milk composition analyzer (HKANGYU KY-9003).

### 2.4 Fecal sample collecting and 16S rRNA gene sequencing

Stool samples were collected from infants at three key time points: 42 days, 6 months, and 12 months postnatally. These timepoints were chosen to span the three major developmental phases of the infant gut microbiota in the First year of life: the initial colonization phase (42 days), the end of the WHO-recommended exclusive breastfeeding period with the transition to complementary foods (6 months), and late infancy, when the microbiota approaches an adult-like structure (12 months). Participants provided infant fecal samples using sterile stool collection tubes, which were promptly transported to the laboratory. Upon arrival, the samples were stored in a −80 °C freezer until further processing.

DNA from stool samples was extracted using the Omega Stool DNA Kit (MoBio Laboratories, Carlsbad, CA) following the manufacturer's protocol. The quality and concentration of the extracted DNA were assessed using 1% agarose gel electrophoresis and spectrophotometry. Post quality assessment, the samples were stored at −20 °C for future analysis. The V3-V4 regions of the 16S rRNA gene were amplified and sequenced on the Illumina MiSeq platform (Illumina, San Diego, CA) at Beijing Ovison Gene Technology Co. Raw sequencing data underwent filtering to remove adapter sequences and low-quality reads. High-quality sequences were clustered into operational taxonomic units (OTUs) using the VSEARCH (v2.7.1) software with the UPARSE algorithm, applying a 97% sequence similarity threshold. Species classification for each OTU was performed by comparing sequences with the Silva128 database using the RDP Classifier algorithm, with a confidence threshold of 70%.

### 2.5 Statistical analysis

Statistical analysis of infant characteristics, breast milk nutrient components, and clinical data was performed using SPSS (version 24.0). Microbiota composition bar plots were generated with R Studio based on species annotation and relative abundance. Alpha-diversity indices (Shannon and Simpson) were calculated in QIIME1 (v1.8.0). Beta-diversity was assessed by principal coordinates analysis (PCoA) based on Bray-Curtis dissimilarity calculated from OTU counts. Differences in microbial community structure among the three timepoints (42 days, 6 months, and 12 months) were tested using permutational multivariate analysis of variance (PERMANOVA) with 999 permutations, performed with the vegan package in R Studio. To examine the relationships between breastfeeding factors and genus-level microbiota composition, multiple linear regression was conducted in R as follows. The outcome was gut microbiota at 12 months of age, with day-42 breast milk composition, maternal diet, and breastfeeding factors as early-life nutritional exposures. Of 16 candidate clinical variables, ts and dqd were excluded *a priori* as deterministic functions of hbs and lbs. Multicollinearity was assessed by variance inflation factors [VIF = 1/(1–R^2^)]. Using an iterative VIF ≥10 exclusion threshold, the variable with the largest VIF was removed and VIFs were recomputed; four breast milk variables were removed in four iterations—water (VIF = 1,402.50), energy (710.22), lactose (120.11), and mineral (24.94)—leaving 10 variables with VIF < 10, ranging from 1.24 (complementary food introduction time) to 5.87 (protein). Forward stepwise regression (by AIC) and LASSO (α = 1) were applied to each retained genus; both ranked feeding mode, breast milk density, and complementary food time as the strongest predictors. Five non-redundant variables (LBS, delivery mode, feeding mode, breast milk density, complementary food introduction time) were retained, with VIFs of 1.13, 1.12, 1.13, 1.10, and 1.03, respectively, indicating negligible multicollinearity; continuous predictors were z-score standardized. Because the infant gut microbiota is highly sparse (median zero proportion ≈ 80%), genera present in ≤ 20% of samples were removed, reducing the number of genera from 412 to 103; remaining zeros were multiplicatively imputed using cmultRepl with CZM and centered log-ratio (CLR) transformed. For each genus, CLR abundance was regressed on the five predictors by ordinary least squares (multiple linear regression, 7 parameters). *P*-values were adjusted for multiple testing using the Benjamini–Hochberg false discovery rate procedure.

As sensitivity analyses, the final regression models were additionally adjusted for parity (primiparous vs. multiparous), gestational age and pre-pregnancy BMI; all associations retained their direction and effect sizes changed by < 20%, indicating that the primary findings were not confounded by these factors.

To minimize potential bias, consecutive enrollment of eligible mother-infant pairs and a follow-up rate >90% were implemented to reduce selection bias. Measurement bias was mitigated by processing all breast milk samples in random order and blinding technicians performing DNA extraction and sequencing to clinical metadata. Missing data (< 5% for any variable) were multiply imputed to limit information bias. All statistical tests were two-sided, with a significance threshold of *P* < 0.05.

## 3 Results

### 3.1 Basic characteristics of study population

**Figure A1:**
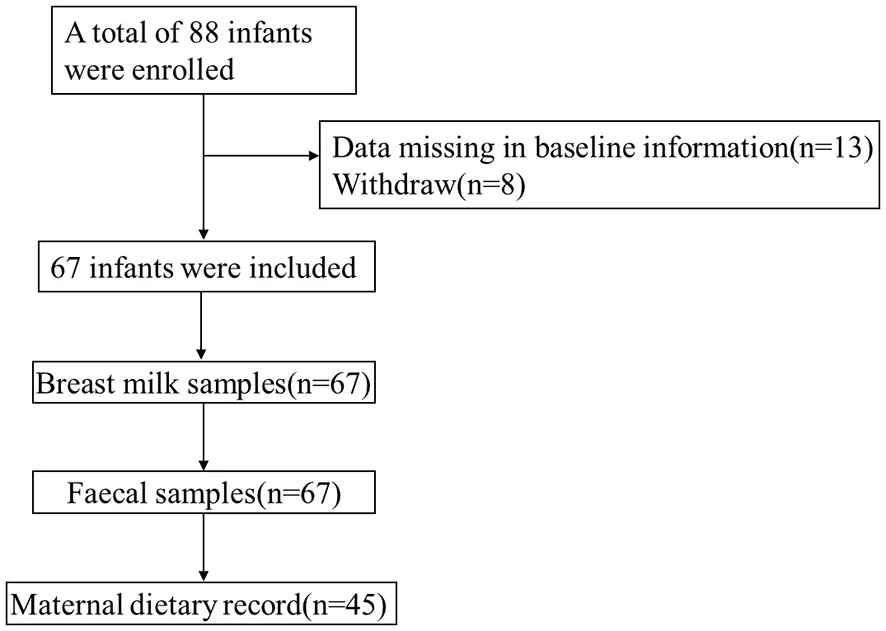
The flowchart of participant enrolment. A total of 88 mother–infant dyads were enrolled. Thirteen were excluded because their baseline information was incomplete: the mother–infant baseline questionnaire, which records maternal age, pre-pregnancy height and weight (BMI), gestational age, parity, delivery mode, feeding mode, and infant birth weight, was missing or not fully completed. A further eight dyads withdrew after enrolment, citing unwillingness to continue the scheduled follow-up visits, relocation to their hometowns for postpartum confinement, or loss to follow-up. The remaining 67 dyads with complete baseline data were included in the analyses. Of the 67 dyads with complete baseline data, complete 3-day dietary records were available for 45 dyads, who were included in the regression analyses.

A total of 67 infants were included in this study, and their baseline characteristics are summarized in Table 1. The enrollment process is detailed in Appendix Figure A1. The mean maternal age was 33.1 ± 3.8 years, and the mean maternal pre-pregnancy BMI was 21.76 ± 3.28 kg/m^2^. The mean gestational age was 38.96 ± 0.99 weeks. Among the mothers, 45 (67.2%) were primiparous and 22 (32.8%) were multiparous. The mean infant birth weight was 3.37 ± 0.36 kg. The cohort comprised 33 male (49.3%) and 34 female (50.7%) infants. Regarding mode of delivery, 38 infants (56.7%) were delivered vaginally and 29 (43.3%) by cesarean section. For feeding practices, 34 infants (50.7%) were exclusively breastfed and 33 (49.3%) were predominantly breastfed. Complementary feeding was introduced at 5 months in 7 infants (10.4%) and at 6 months in 60 infants (89.6%).

**Table 1:** Basic characteristics of study population.

Characteristic	Value
Total infants	67
Maternal age (years)	33.1 ± 3.8
Maternal pre-pregnancy BMI (kg/m^2^)	21.76 ± 3.28
Gestational age (weeks)	38.96 ± 0.99
Birth weight (kg)	3.37 ± 0.36
Parity	
Primiparous	45 (67.2%)
Multiparous	22 (32.8%)
Gender	
Male	33 (49.3%)
Female	34 (50.7%)
Mode of delivery	
Vaginal birth	38 (56.7%)
Cesarean section	29 (43.3%)
Feeding practices	
Exclusive breastfeeding	34 (50.7%)
Predominant breastfeeding	33 (49.3%)
Complementary food time	
5 months	7 (10.4%)
6 months	60 (89.6%)

### 3.2 Breastfeeding factors

Key factors associated with breastfeeding include mothers' dietary patterns, breast milk composition and duration of breastfeeding. These factors provide a comprehensive view of the nutritional status and breastfeeding practices of lactating mothers and are critical to understanding their potential impact on the health of their infants.

#### 3.2.1 Evaluation of the dietary patterns

The dietary structure of lactating mothers was assessed through a detailed questionnaire, which captured the intake amount and frequency of different food types. Using this data, the average daily intake of various foods was estimated. The dietary status was then quantified using the DBI scoring method (Table 2).

**Table 2:** Dietary intake and scores.

Characteristic	Daily intake (g)	Assigned value
Cereals and yams	234.3 (172.4, 306.6)	0 (0, 0)
Vegetables	345.7 (225.6, 470.1)	−1 (−3, 0)
Fruit	203.6 (119.8, 344.5)	−2 (−3.25, 0)
Dairy	171.4 (61.4, 266.4)	−2.5 (−4.25, −0.75)
Beans	26.5 (5.1, 76.3)	0 (−2.25, 0)
Meat	105.5 (71.5, 177.4)	3 (1, 4)
Fish	19.3 (7.1, 42.9)	−2.5 (−3, −1)
Eggs	77.1 (50.9, 114.6)	2 (1, 3)
Oil	20.0 (16.9, 22.9)	0 (0, 1)
Alcohol	0.0 (0, 0)	0 (0, 0)
Salt	6.0 (5, 7)	0 (0, 0)
Sugar	0.0 (0, 0)	1 (0, 1)
Water	1,750.0 (1,250.0, 2,000.0)	0 (−3, 0)

The estimated daily energy intake for the study population ranged from 1,875 to 2,075 kcal, with a median of 2,000 kcal. The DBI scores for the participants were as follows: the HBS was 6 (4, 8), indicating the degree of excessive intake; the LBS was −10 (−17, −7), reflecting the extent of dietary deficiencies; the TS was −5.5 (−11.5, 0), representing the overall dietary quality; and the DQD was 18.5 (14, 25), highlighting the imbalance in the intake of various food categories.

#### 3.2.2 Breast milk composition

The composition of breast milk was analyzed in all 67 mothers; a single mid-feed spot sample was collected from each mother at 42 days postpartum (Section 2.3). The average fat content in the breast milk was 3.08 ± 1.3 g/100 ml. Protein content averaged 0.68 ± 0.30 g/100 ml. Lactose was found to be 5.3 ± 2.3 g/100 ml. The energy content of the breast milk was measured at 51.9 ± 17.8 kcal/100 ml. The water content averaged 90.7 ± 3.3%, and the mineral content was 0.16 ± 0.07 g/100 ml. Finally, the density of the breast milk was found to be 1.02 ± 0.01 g/ml. These measurements provide essential data for understanding the nutritional quality of breast milk and its potential impact on infant health.

#### 3.2.3 Duration of breastfeeding

The duration of breastfeeding among the study participants was assessed. On average, mothers breastfed their infants for 4.9 months. Notably, 52% of the mothers continued breastfeeding for more than 6 months.

### 3.3 Characteristics and temporal changes of infant gut microbiota

**Figure 1:**
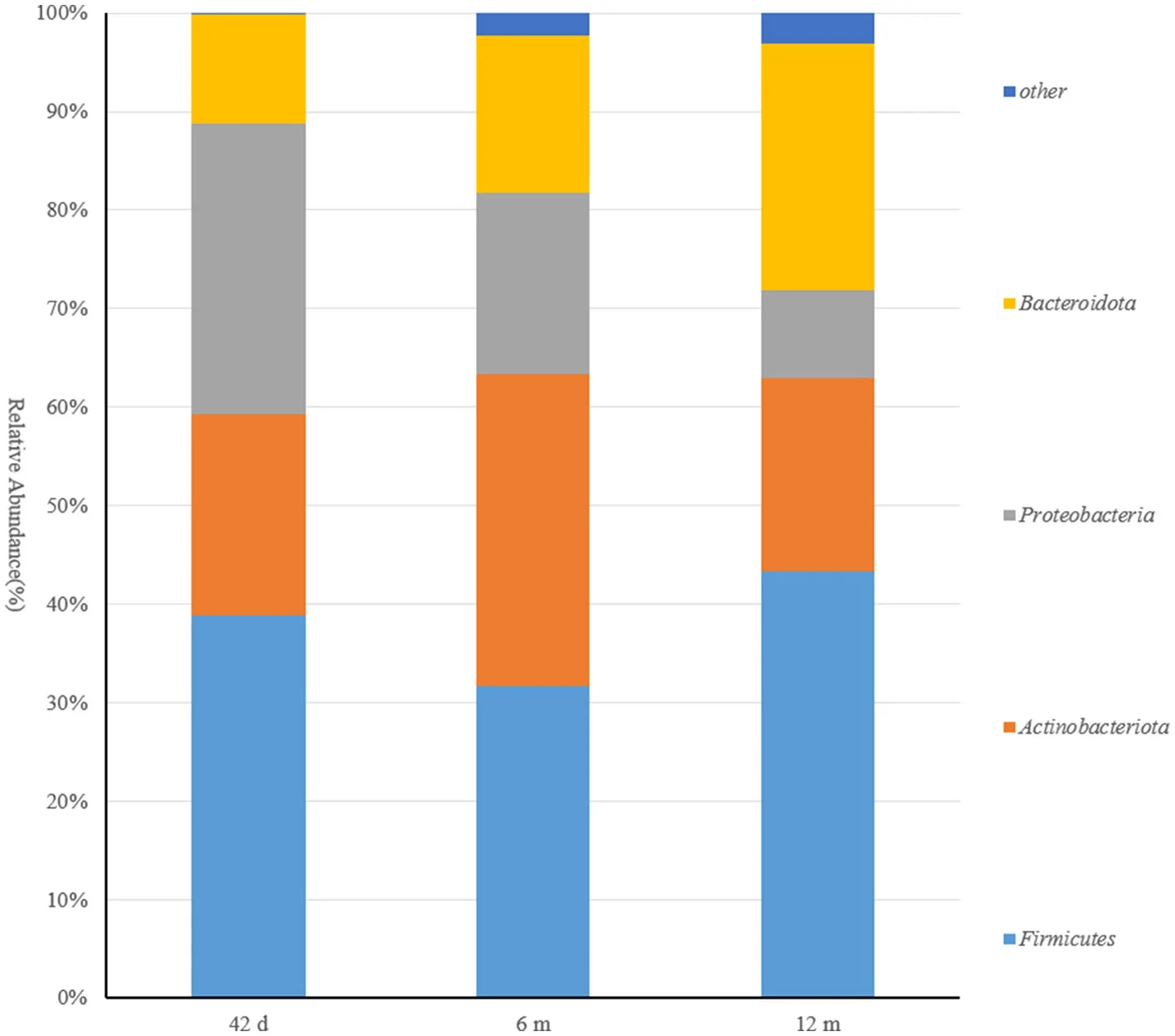
Temporal changes in phylum-level gut microbiota composition in infants. Stacked bar chart showing the mean relative abundance (%) of gut microbiota phyla in infant fecal samples at 42 days, 6 months, and 12 months. Each bar represents the mean across all infants at the corresponding time point, and different color segments represent individual phyla as indicated in the legend.

The relative abundance of gut microbiota at the phylum level in infants was analyzed at three key time points: 42 days, 6 months, and 12 months (Figure 1).

At the phylum level, *Firmicutes* initially represented 38.8% of the gut microbiota at 42 days, decreased to 31.6% at 6 months, and then increased to 43.4% by 12 months. *Actinobacteriota* showed a substantial increase from 20.5% at 42 days to 31.8% at 6 months, before decreasing to 19.7% at 12 months. *Proteobacteria* exhibited a marked decline over time, starting at 29.5% at 42 days, dropping to 18.4% at 6 months, and further reducing to 8.9% at 12 months. Conversely, *Bacteroidota* showed a consistent increase, starting at 11.1% at 42 days, rising to 15.9% at 6 months, and reaching 25.1% by 12 months. The proportion of other phyla also increased slightly.

**Figure 2:**
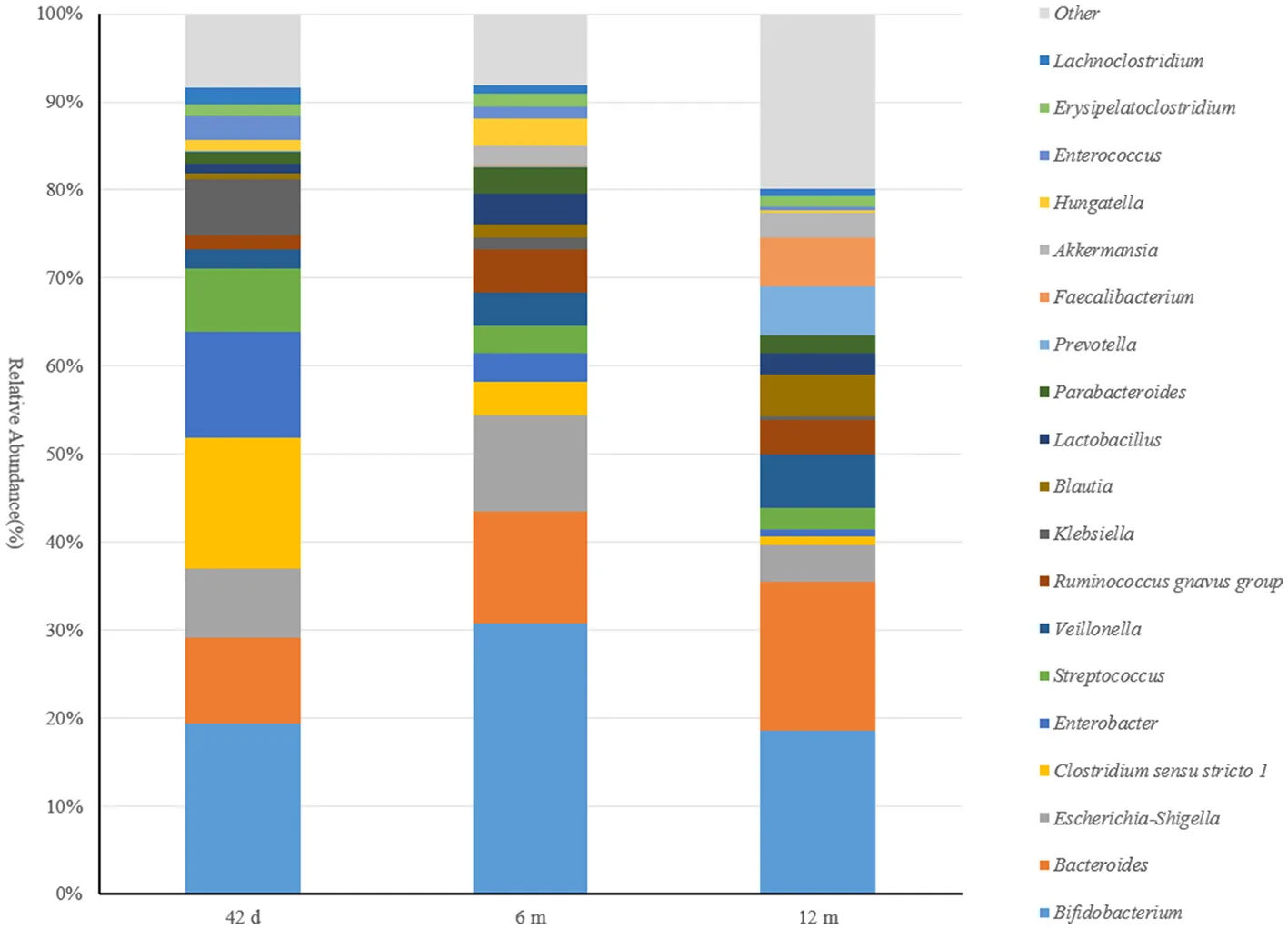
Temporal changes in genus-level gut microbiota composition in infants. Stacked bar chart showing the mean relative abundance (%) of gut microbiota genera in infant fecal samples at 42 days, 6 months, and 12 months. Each bar represents the mean across all infants at the corresponding time point, and different color segments represent individual genera as indicated in the legend.

The data on phylum-level changes highlight significant shifts in the gut microbiota composition over the First year of life. To further understand these dynamics, the relative abundance at the genus level was also examined at 42 days, 6 months, and 12 months (Figure 2).

At 42 days, the dominant genera included *Bifidobacterium* (19.4%), *Clostridium sensu stricto 1* (14.9%), *Enterobacter* (12.0%), and *Bacteroides* (9.6%). As the infants aged to 6 months, a notable increase in *Bifidobacterium* (30.7%) and *Bacteroides* (12.8%) was observed, while the abundance of *Clostridium sensu stricto 1* (3.8%) and *Enterobacter* (3.2%) significantly decreased. By 12 months, *Bifidobacterium* remained one of the most prevalent genera at 18.5%, but *Bacteroides* increased further to 17.0%, indicating its growing significance in the gut microbiota. There was also a rise in the relative abundance of *Veillonella* (6.1%) and *Prevotella* (5.6%), with the latter not being present at earlier stages. Conversely, *Escherichia-Shigella* and *Enterobacter* showed substantial reductions in their relative abundance, down to 4.1% and 0.9% respectively.

### 3.4 Temporal changes in alpha and beta diversity of infant gut microbiota

**Figure 3:**
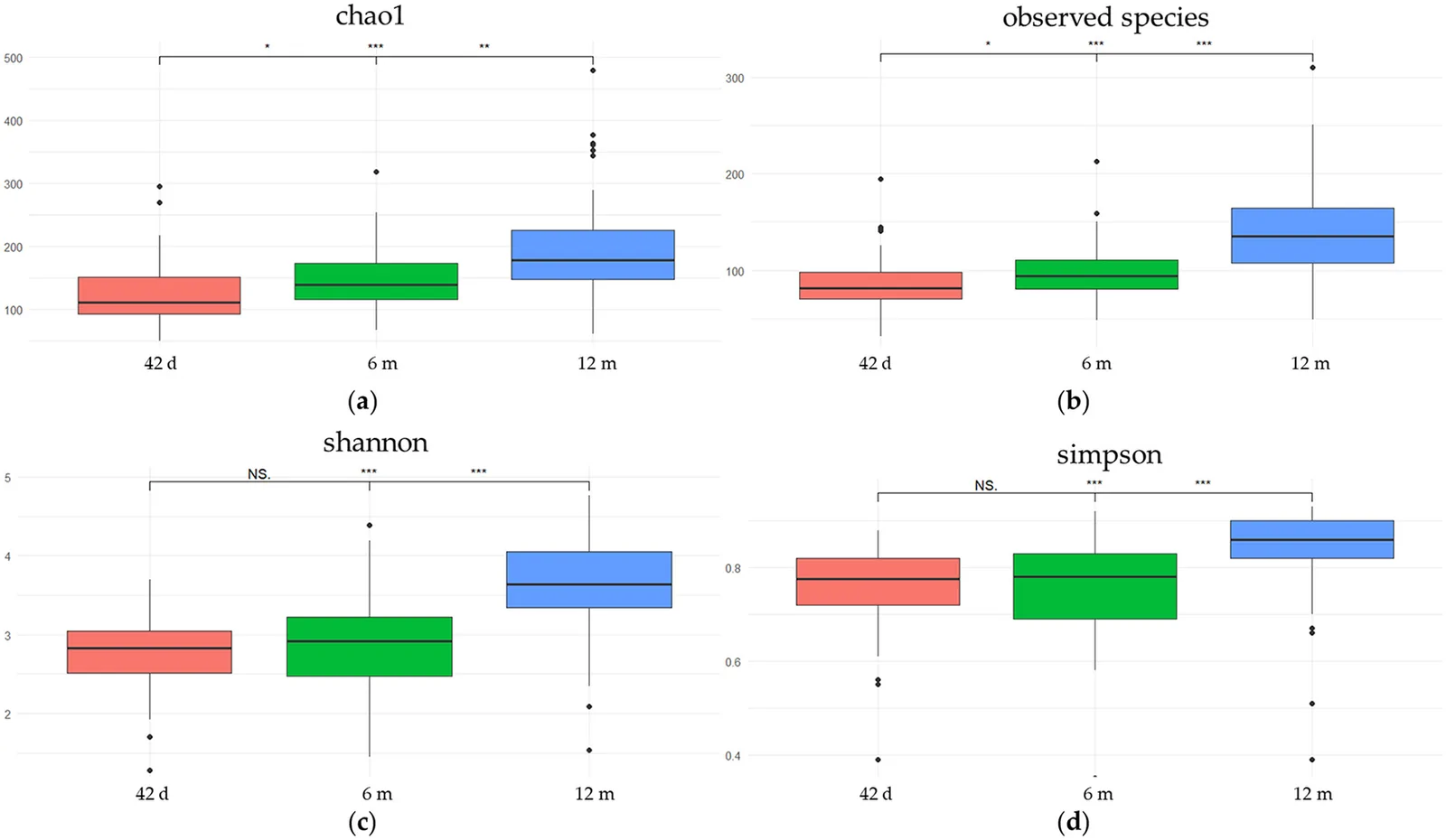
Alpha diversity indices of infant gut microbiota at 42 Days, 6 Months, and 12 Months. Box plots show the distribution of **(a)** Chao1 index, **(b)** observed species, **(c)** Shannon index, and **(d)** Simpson index at each time point. In each box plot, the horizontal line inside the box represents the median, the box boundaries the interquartile range, and the whiskers the extent of the data. Statistical significance between time points is indicated as follows: “*” represents *p* < 0.05, “**” represents *p* < 0.01, “***” represents *p* < 0.001, and “NS” stands for not significant.

The alpha diversity of the infant gut microbiota at 42 days, 6 months, and 12 months was assessed using several diversity indices: Chao1, observed species, Shannon, and Simpson indices. The results, depicted in the boxplots (Figure 3), indicate significant changes over time. The Chao1 index, which estimates the richness of the microbiota, shows a significant increase from 42 days to 6 months (*p* < 0.05), from 42 days to 12 months (*p* < 0.001), and from 6 months to 12 months (*p* < 0.01). Similarly, the number of observed species, reflecting the actual count of distinct species, significantly increases from 42 days to 6 months (*p* < 0.05), from 42 days to 12 months (*p* < 0.001), and from 6 months to 12 months (*p* < 0.001).

The Shannon index, which accounts for both richness and evenness, shows no significant change from 42 days to 6 months. However, it significantly increases from 42 days to 12 months (*p* < 0.001) and from 6 months to 12 months (*p* < 0.001), indicating a more even distribution of species over time. The Simpson index, which measures diversity with an emphasis on common species, shows no significant difference between 42 days and 6 months but exhibits a significant increase from 42 days to 12 months (*p* < 0.001) and from 6 months to 12 months (*p* < 0.001). These results suggest that the alpha diversity of the infant gut microbiota increases significantly as the infants grow, particularly between 6 and 12 months, reflecting a more complex and diverse microbial community with age.

**Figure 4:**
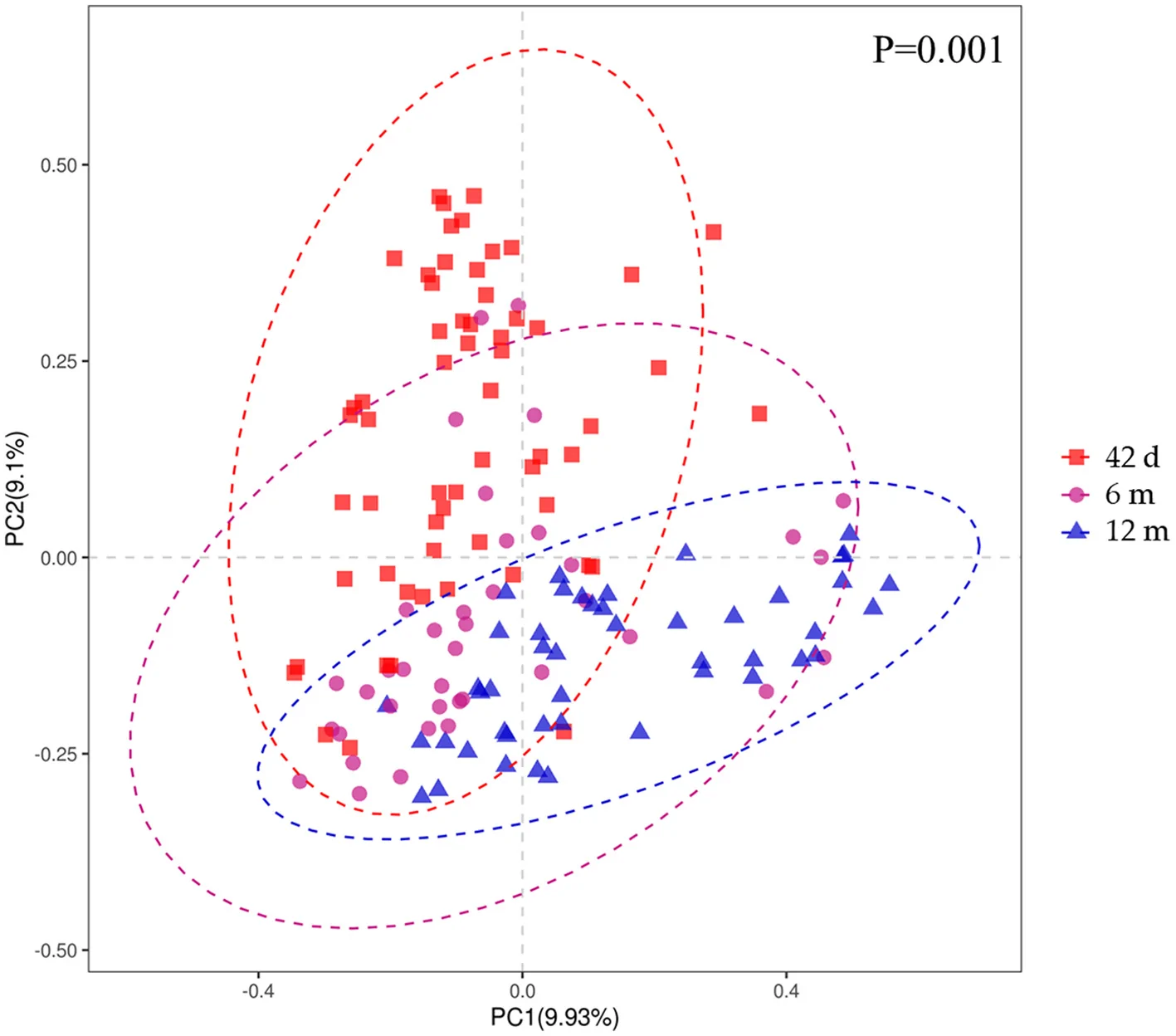
Beta diversity of infant gut microbiota at 42 days, 6 months, and 12 months. Principal coordinates analysis (PCoA) plot based on Bray–Curtis dissimilarity calculated from OTU counts; each point represents one infant fecal sample. Red squares, pink circles, and blue triangles represent samples collected at 42 days, 6 months, and 12 months, respectively. Differences in microbial community composition among the three time points were assessed by permutational multivariate analysis of variance (PERMANOVA) with 999 permutations (*P* = 0.001).

The beta diversity of infant gut microbiota at 42 days, 6 months, and 12 months was analyzed using Principal Coordinates Analysis (PCoA) using Bray-Curtis distance based on the OTU counts. The PCoA plot (Figure 4) shows the clustering of microbiota samples based on their composition at the three different time points. Each point represents a sample, with red squares indicating samples at 42 days, pink circles for 6 months, and blue triangles for 12 months. The observed clustering and statistical significance (*P* = 0.001) indicate that the gut microbiota undergoes significant changes during the First year of life, with distinct microbial communities forming at each developmental stage. This analysis underscores the dynamic nature of the infant gut microbiome and its evolution over time.

### 3.5 Multiple linear regression analysis of breastfeeding factors and gut microbiota

To identify breastfeeding-related factors associated with the infant gut microbiota at 12 months of age, multiple linear regression was performed in 45 mother–infant dyads with complete dietary data. After exclusion of perfectly collinear derived variables, iterative variance-inflation-factor (VIF) screening and feature selection (forward stepwise regression by AIC and LASSO), five non-redundant predictors were retained: the maternal dietary Low Bound Score (LBS), delivery mode, feeding mode, breast milk density and complementary food introduction time; continuous predictors were z-score standardized (VIF 1.03–1.13). Of the 412 genera initially annotated, genera present in ≤ 20% of samples were removed (*n* = 103); remaining zeros were replaced multiplicatively (cmultRepl, CZM) and abundances were CLR transformed. Each genus was then regressed on the five predictors by ordinary least squares (7 parameters), and *p*-values were corrected using the Benjamini–Hochberg false discovery rate (FDR) procedure within each genus. Of the 618 coefficient-level tests, 14 associations remained significant at q < 0.05 (Table 3). No association survived FDR correction at the model level or globally across all coefficients, consistent with the number of false positives expected by chance; the 14 associations nevertheless clustered around feeding mode (8 of 14).

**Table 3:** Multivariable linear regression associations of gut microbial relative abundances at phylum and genus levels with feeding practices and other predictors.

Taxon	Variable	β (SE)	*t*	*p*	q (FDR)
*Agathobacter*	Feeding	+2.195 (0.590)	3.72	0.0006	0.0031
*Roseburia*	Feeding	+2.137 (0.672)	3.18	0.0029	0.0145
*Tyzzerella*	Feeding	+2.126 (0.657)	3.23	0.0025	0.0124
*Neisseria*	Feeding	−0.757 (0.279)	−2.71	0.0100	0.0498
*Rothia*	Feeding	−0.541 (0.196)	−2.77	0.0086	0.0269
*Fusobacterium*	Feeding	−1.048 (0.403)	−2.60	0.0131	0.0438
*Campylobacter*	Feeding	−0.973 (0.328)	−2.96	0.0052	0.0258
*Phylum Campilobacterota*	Feeding	−0.973 (0.328)	−2.96	0.0052	0.0258
*Rothia*	Delivery	+0.629 (0.235)	2.68	0.0108	0.0269
*Ruminococcus gnavus group*	Delivery	−1.767 (0.573)	−3.08	0.0038	0.0188
*Staphylococcus*	Complementary food time	−0.445 (0.163)	−2.72	0.0096	0.0481
*Fusobacterium*	Complementary food time	−0.483 (0.195)	−2.48	0.0175	0.0438
*Agathobacter*	LBS	−0.794 (0.298)	−2.66	0.0113	0.0282
*Phylum Bacteroidota*	Density	+0.824 (0.304)	2.71	0.0099	0.0495

SE, standard error; FDR, false discovery rate; CLR, centered log-ratio. Values are β coefficients, SE, *t*, raw *p*, and FDR-adjusted q. Feeding mode: mixed formula-fed vs. exclusively breastfed (reference); delivery mode: cesarean vs. vaginal delivery (reference); complementary food time, LBS (maternal dietary Low Bound Score), and breast milk density were z-standardized. q < 0.05 indicates significance after FDR correction.

Feeding mode was the dominant predictor. Relative to exclusively breastfed infants, mixed breastfed infants showed significantly higher CLR abundances of *Agathobacter* (β = 2.195, q = 0.0031), *Roseburia* (β = 2.137, q = 0.0145) and *Tyzzerella* (β = 2.126, q = 0.0124), and significantly lower abundances of *Campylobacter* (β = −0.973, q = 0.0258), *Rothia* (β = −0.541, q = 0.0269), *Neisseria* (β = −0.757, q = 0.0498) and *Fusobacterium* (β = −1.048, q = 0.0438). The association for the phylum *Campilobacterota* (β = −0.973) was identical to that of its only detected genus *Campylobacter* and is therefore not considered a separate finding.

Delivery mode was associated with two genera. Relative to vaginal delivery, cesarean section was associated with a higher abundance of *Rothia* (β = 0.629, q = 0.0269) and a lower abundance of the *Ruminococcus gnavus group* (β = −1.767, q = 0.0188). Complementary food introduction time was negatively associated with *Staphylococcus* (β = −0.445, q = 0.0481) and *Fusobacterium* (β = −0.483, q = 0.0438). As most infants (40/45, 88.9%) were introduced to complementary foods at 6 months and the remaining 5 (11.1%) at 5 months, these associations indicate that the small group introduced at 5 months carried higher abundances of both genera. The maternal dietary LBS, which reflects the extent of dietary deficiency, was negatively associated with *Agathobacter* abundance (β = −0.794, q = 0.0282), indicating higher abundance of this genus in infants whose mothers reported more pronounced dietary deficiencies. Breast milk density was positively associated with the abundance of the phylum *Bacteroidota* (β = 0.824, q = 0.0495), indicating higher *Bacteroidota* abundance in infants of mothers with denser breast milk.

## 4 Discussion

The study population of 67 infants, with nearly equal gender distribution, varied birth weights, and a mix of vaginal and cesarean deliveries, provides a robust sample for analyzing the impact of breastfeeding on gut microbiota. The high rate of exclusive and predominant breastfeeding among participants supports the study's aim to investigate the effects of breastfeeding practices on microbiota development.

Breastfeeding-related factors, including maternal dietary patterns, breast milk composition, feeding mode, delivery mode and complementary food introduction time, are critical to understanding the potential impact of breastfeeding practices on infant health.

Despite 52% of mothers breastfeeding for more than 6 months, the overall average duration of breastfeeding was 4.9 months. This discrepancy suggests that a considerable portion of the remaining 48% breastfed for significantly shorter periods, thereby lowering the overall average duration. This variation underscores the diversity in breastfeeding practices within the study population.

Promoting a proper diet for breastfeeding mothers ensures that the nutritional content of breast milk is optimized (8). One of the distinctive features of this study is the quantitative evaluation of the dietary patterns of lactating mothers against the Chinese Dietary Guidelines. This study highlights the importance of aligning maternal dietary practices with established dietary guidelines to improve breastfeeding outcomes. By demonstrating the connection between maternal diet and infant gut microbiota, it provides a clear direction for promoting breastfeeding through targeted nutritional education and support. These findings reinforce the need for comprehensive dietary guidance and support for lactating mothers to ensure the best possible health outcomes for both mothers and their infants.

The HBS reflects the overconsumption of certain foods. The data suggest that the participants, on average, consumed meat, eggs, and sugar in excess of their recommended daily allowances. This may be linked to traditional Chinese beliefs that encourage the consumption of foods like eggs and brown sugar water during the breastfeeding period to boost health and milk production (9, 10). Such practices are rooted in the cultural emphasis on ensuring the mother's recovery and the baby's health through specific dietary measures. The LBS, on the other hand, points to deficiencies in the intake of fish, dairy products, vegetables, and fruits. This deficiency might be influenced by regional dietary habits, as Beijing, being inland, has a lower propensity for fish consumption compared to coastal areas (11). Additionally, there may be a common misconception among lactating women that dairy consumption is not necessary, leading to reduced intake of dairy products. The insufficient intake of dairy products among lactating mothers can result from a complex interplay of cultural beliefs, Lactose Intolerance, dietary preferences, lack of education, and socioeconomic factors. Vegetables and fruits are also often underconsumed due to dietary preferences and convenience. The DQD was relatively large, indicating significant dietary imbalances and the presence of picky eating habits among the participants. This dietary imbalance can lead to uneven nutrient intake, potentially affecting the quality of breast milk and, consequently, infant health.

The composition of breast milk was found to vary among the participants. These components are essential for providing the necessary nutrients for infant growth and development. Breast milk is essential for the development of intestinal flora in newborns (12).

Analysis of infant intestinal flora shows that the infant gut microbiome undergoes significant compositional changes over time. Early life microbiota is characterized by higher levels of *Proteobacteria* and *Actinobacteriota*, which decrease as the infant ages, while *Firmicutes* and *Bacteroidota* become more dominant, reflecting the maturation and diversification of the gut microbiome during the First year of life.

For the case of genus level, initially dominated by a few key genera, the microbiota becomes more diverse with the emergence and increase of previously less prevalent or absent genera such as *Faecalibacterium* and *Prevotella* by 12 months. The proportion of unclassified or other genera also increases significantly from 8.3% at 42 days to 19.9% at 12 months, reflecting the growing complexity and diversity of the gut microbiome as the infants develop. Large longitudinal studies have similarly shown that infant age explains a major proportion of gut microbiome variation and that the community progresses from an early milk-adapted state toward a more diverse configuration as complementary and family foods are introduced (13, 14). Shotgun metagenomic data further indicate that birth-related differences can define distinct developmental trajectories through the First year, even while the overall microbiota converges toward a more adult-like taxonomic profile (15).

Feeding mode accounted for the largest share of the exploratory associations. Compared with the exclusively breastfed reference group, predominantly breastfed infants had higher CLR abundances of *Agathobacter, Roseburia*, and *Tyzzerella*. These taxa are frequently detected during the expansion of *Lachnospiraceae*-associated communities that accompanies dietary diversification; however, their presence should not be equated with a uniformly beneficial microbiota. Studies of the complementary feeding period show that the transition to protein- and fiber-containing family foods is closely associated with increased alpha diversity and enrichment of adult-associated fermentative taxa (16). Conversely, continued breastfeeding can maintain a more *Bifidobacterium*-rich and less adult-like community beyond the period of exclusive milk feeding (17, 18). The higher abundances of the three taxa in the predominantly breastfed group may therefore reflect a faster transition toward a complementary-food-associated state rather than a simple improvement or deterioration in gut health. These feeding-mode associations persisted after adjustment for complementary food introduction time, and they parallel the concurrent rise in alpha diversity and the progressive separation of the 12-month community from the earlier time points in the present cohort.

The association of feeding mode with *Roseburia* is also compatible with a prospective birth-cohort study in which earlier complementary feeding was associated with a *Roseburia* amplicon sequence variant and higher fecal butyrate at 12 months (16). Nevertheless, the present study measured taxonomic composition rather than microbial genes or metabolites; consequently, short-chain-fatty-acid production cannot be inferred from genus-level relative abundance. This distinction is particularly important for *Tyzzerella* and other taxonomically broad genera whose strains may differ substan-tially in metabolic function.

The predominantly breastfed group also had lower CLR abundances of *Campylobacter, Rothia, Neisseria*, and *Fusobacterium*. Several of these genera are common in the oral cavity, and strain-resolved mother-infant studies have demonstrated substantial sharing between infant oral and gut communities, including transmission of *Rothia* strains (19). Their detection in feces may therefore reflect oral-gut dispersal, feeding behavior, and community maturation rather than intestinal pathogenicity. A GEMS analysis reported relatively high *Campylobacter* abundance among exclusively breast-fed infants, but its strongest association occurred in infants with diarrhea and its healthy-control comparison was not statistically significant (20). That study offers directional context but is not directly comparable with this healthy Beijing cohort. Species- or strain-resolved sequencing and clinical phenotyping would be required before assigning pathogenic significance to these genus-level differences.

Delivery mode was associated with higher *Rothia* and lower *Ruminococcus gnavus* group abundance after cesarean delivery. Cesarean birth can disrupt maternal gut microbial transmission and favor early acquisition of skin-, oral-, or hospital-associated organisms (21). In large cohorts, delivery mode has also been associated with differences in *Bacteroides* colonization and with microbiota trajectories that may persist to 12 months (22). The *R. gnavus* group is a frequent early member of the infant gut microbiota (23). Its lower abundance after cesarean delivery is therefore biologically plausible, but published birth-mode effects are heterogeneous and may be modified by intrapartum antibiotics, breastfeeding, and later diet. The opposite directions of the feeding-mode and delivery-mode coefficients for *Rothia* further illustrate that no single taxon should be interpreted without considering the broader exposure context.

Complementary food introduction time was negatively associated with *Staphylococcus* and *Fusobacterium*, indicating higher abundances in the five infants introduced to complementary foods at 5 months than in the 40 introduced at 6 months. Complementary feeding is a recognized ecological transition that changes both microbiota composition and microbial metabolite production (16). However, the one-month contrast in the present cohort was highly unbalanced, and the types, diversity, and quantities of foods were not incorporated into the model. The associations may conse-quently reflect other differences between the two small groups, including breastfeeding status, illness, antibiotic exposure, or the composition of complementary foods. These results should not be used to infer that introducing complementary foods at 5 or 6 months causes an increase or decrease in either genus, nor do they support changing current infant-feeding recommendations.

Maternal dietary LBS was negatively associated with infant *Agathobacter* abundance. Under the present LBS coding, this coefficient was interpreted as a higher abundance among infants of mothers with greater dietary deficiency. This direction should not be construed as evidence that maternal dietary inadequacy is beneficial. One possibility is that very low LBS values, in this study context, coincide with high consumption of refined carbohydrate staples (e.g., rice and wheat-based foods), and this dietary pattern may indirectly influence the infant gut environment through alterations in breast milk macronutrient profiles. Previous studies have shown that maternal diet can influence milk fatty-acid and oligosaccharide profiles, and paired mother-infant studies provide plausible pathways linking maternal intake, milk com-position, and the early infant microbiota (24, 25). However, because human milk oligosaccharides or fatty acid profiles were not directly measured in the present study, this interpretation remains speculative. Moreover, the current association has not been established previously for *Agathobacter*, and residual socioeconomic, household, or post-weaning dietary factors could explain the finding. Replication with repeated ma-ternal dietary measurements and mediation analysis of specific milk components is needed.

Breast milk density at 42 days was positively associated with *Bacteroidota* abundance at 12 months. Human milk composition and milk-associated microbial communities have been linked with infant fecal microbiota in prospective studies (26), but density is a composite physical measure rather than a single bioactive nutrient. In addition, *Bacteroidota* and particularly *Bacteroides* show strong age-, feeding-, and birth-mode-dependent trajectories during the First year (15). The observed coefficient may therefore represent an early nutritional marker or correlated milk property rather than a direct effect of milk density itself. Its borderline within-taxon q value, the use of one mid-feed spot sample, and the long interval between milk and fecal sampling warrant conservative interpretation.

This study has several strengths. The same cohort was assessed at three biologically informative stages spanning predominantly milk feeding, complementary feeding, and the transition toward a family diet. Maternal dietary information, breast milk composition, and key perinatal and feeding variables were considered together. In the present analysis, use of a common 12-month outcome removed age as a within-model confounder; highly collinear derived and milk variables were excluded, retained predictors had low VIF values, sparse taxa were filtered, and compositional abundance data were CLR transformed. Sensitivity analyses that additionally included parity, gestational age, and prepregnancy BMI produced similar directions and effect sizes, supporting the stability of the reported coefficients within this dataset.

Several limitations remain. First, the regression analysis included only 45 dyads and fitted seven parameters, limiting statistical power and increasing the risk of unstable taxon-specific estimates. Second, the 16S rRNA approach provides limited species-, strain-, and functional resolution and cannot determine absolute bacterial loads. Third, maternal diet and breast milk composition were measured early in lactation, whereas microbiota outcomes were measured at 12 months, when current breastfeeding status and complementary or family foods are likely to be major determinants. Detailed longitudinal data on breastfeeding cessation, complementary food composition, infections, antibiotics, probiotics, siblings, daycare, and other household exposures were unavailable or not included. Fourth, the complementary-feeding comparison was markedly imbalanced, and a single milk sample cannot represent changes across lactation. Fifth, infant sex was not retained in the final multivariable models, and potential sex-related differences in infant microbiota development could therefore not be evaluated. Finally, the observational design precludes causal inference. Larger studies should combine repeated exposure and microbiome measurements in mixed-effects models, use shotgun metagenomics and metabolomics, and prespecify global multiple-testing control.

In conclusion, the data support a model in which infant gut microbiota at 12 months reflects the combined influence of feeding pattern, delivery mode, complementary feeding, and early maternal-milk factors against a dominant background of age-related ecological maturation. Feeding mode generated the most consistent cluster of taxon-level signals, but the findings do not establish that any single feeding practice, maternal dietary score, or milk characteristic caused a healthier or less healthy micro-biota. The identified associations provide specific hypotheses for validation in larger, well-powered longitudinal cohorts.

## 5 Conclusions

This study characterized longitudinal changes in the infant gut microbiota of a Chinese birth cohort across three time points in the First year of life. Phylum-level composition shifted markedly, with increases in *Firmicutes* and *Bacteroidota* and decreases in *Actinobacteriota* and *Proteobacteria* from 42 days to 12 months, accompanied by increasing alpha diversity and by clear time-point separation of the 12-month community in beta diversity analysis. At 12 months, feeding mode was the dominant predictor of taxon abundance, and predominantly breastfed infants differed from exclusively breastfed infants in the abundance of seven genera. Delivery mode, complementary food introduction time, and maternal dietary LBS were each associated with smaller sets of taxa, and higher breast milk density at 42 days was positively associated with *Bacteroidota* abundance at 12 months.

These associations are exploratory and require confirmation in larger longitudinal cohorts with repeated exposure measurements and strain- or species-level resolution. Nevertheless, the findings support current infant-feeding recommendations and suggest that the developing infant gut microbiota is shaped jointly by feeding practice, delivery mode, complementary feeding, and early maternal milk characteristics.

## Data Availability

The data analyzed in this study is available in the NCBI repository (https://www.ncbi.nlm.nih.gov) under BioProject accession number PRJNA1195335.
